# Mechanisms of Negative Fetal Outcome in Frontal Vehicle Collisions Involving Unbelted Pregnant Drivers

**DOI:** 10.3390/healthcare9010025

**Published:** 2020-12-29

**Authors:** Arisa Takeda, Yasuki Motozawa, Marin Takaso, Mami Nakamura, Shinobu Hattori, Masahito Hitosugi

**Affiliations:** 1 Department of Legal Medicine, Shiga University of Medical Science, Otsu, Shiga 520-2192, Japan; marint@belle.shiga-med.ac.jp (M.T.); mamin@belle.shiga-med.ac.jp (M.N.); shattori@fujita-hu.ac.jp (S.H.); hitosugi@belle.shiga-med.ac.jp (M.H.); 2 Department of Mechanical and Precision System, Teikyo University, Utsunomiya, Tochigi 320-8551, Japan; y.motozawa@mps.teikyo-u.ac.jp

**Keywords:** pregnant women, frontal motor vehicle collisions, negative fetal outcome

## Abstract

To determine the cause of negative fetal outcomes and the causative mechanism in a frontal collision, we analyzed the kinematics and mechanisms of injuries using an unbelted pregnant dummy, the Maternal Anthropometric Measurement Apparatus dummy, version 2B. Sled tests were performed to recreate frontal impact situations with impact speeds of 13, 26, and 40 km/h. Overall kinematics of the dummy were examined through high-speed video imaging. Quantitative dummy responses—such as time courses of the abdominal pressure, chest deflection, neck injury criteria (Nij), and displacement of the pelvis during impact—were also measured. The maximum abdominal pressure of 103.3 kPa was obtained at an impact speed of 13 km/h. The maximum chest deflection of 38.5 mm and Nij of 0.36 were obtained at an impact speed of 26 km/h. The highest maximum chest deflection of >40.9 mm, Nij of 0.61, and forward pelvis displacement of 478 mm were obtained at an impact speed of 40 km/h. Although the kinematics and mechanism of injuries of the dummy were different for different collision speeds, we found that unbelted pregnant drivers suffer severe or fatal injuries to the fetus even in low-speed collisions.

## 1. Introduction

Road traffic injury is a major public issue worldwide. According to the World Health Organization, approximately 1.35 million people die each year as a result of road traffic collisions in 2018 [1]. Pregnant women are also often involved in motor vehicle collisions (MVCs), potentially leading to negative fetal outcomes. Klinich et al. reported that approximately 130,000 women in late-term pregnancy are involved in MVCs in the United States annually, and the annual estimate of abortions or stillbirths ranges from 300 to 3800 [2]. Kvarnstrand et al. reported that the incidence rates of maternal and fetal deaths related to MVCs were respectively 1.4 and 3.7 per 100,000 pregnancies in Sweden [3]. These reports indicate a worldwide need to clarify the injury mechanisms of pregnant drivers and to reduce the rates of fatalities of pregnant women and their fetuses.

The wearing of three-point seatbelts is effective in terms of reducing the fatality rate and severity of motor vehicle injuries. Previous reports on actual MVCs cases found that the wearing of a seatbelt by pregnant drivers reduces both the maternal injury severity and fetal fatality rate, in contradistinction to not wearing a seatbelt [4,5]. The American College of Obstetricians and Gynecologists therefore recommended wearing a seatbelt correctly for reducing the risk of injury to both the mother and fetus [6]. From the biomechanical viewpoint, positive effects of three-point seatbelts for pregnant women involved in frontal and rear-end collisions were confirmed with an anthropometric model of a pregnant woman [7,8]. The cited studies found that wearing a seatbelt reduced abdominal pressure or prevented contact with the steering wheel during a collision. However, a substantial number of pregnant women are not willing to wear three-point seatbelts because of a compressive feeling or discomfort. Acar et al. suggested that the rate of seatbelt use during pregnancy in Europe, North America, and other countries was 94.8%, 92.2%, and 83.3%, respectively [9]. In Malaysia, 57.6% of pregnant drivers always used a seatbelt [10]. Even in countries such as the United States, where the wearing of seatbelts is legally required, more than 20% of pregnant women do not wear seatbelts [5,11,12]. To prevent the negative fetal outcomes, the cause of fetal death and mechanisms of fatal injuries when an unbelted pregnant driver is involved in a frontal collision should be enlightened. However, to the best of our knowledge, there have been no reports on the kinematics and affected biomechanical parameters of unbelted pregnant vehicle passengers during a collision.

To determine the cause of the negative fetal outcome and causative mechanism in a frontal collision involving an unbelted pregnant driver, we performed biomechanical analyses using a dummy of a pregnant woman.

## 2. Materials and Methods

### 2.1. Dummy

The dummy used herein is the most current version of the Maternal Anthropometric Measurement Apparatus, version 2B (MAMA-2B), developed by First Technology Safety Systems and the University of Michigan Transportation Research Institute in 2001 [13,14,15]. This dummy is based on the Hybrid-III American Female fifth percentile (AF05) dummy and is thus suitable for vehicle impact tests and for analysis of the kinematics of pregnant women during MVCs. The pelvis, sternum, and ribcage are modified to accommodate a silicone rubber bladder representing the uterus at 30 weeks of gestation. The size of the dummy is based on an American woman in the fifth percentile; i.e., a small woman with a height of 153 cm. This size is in accordance with a standard Japanese pregnant woman at 30 weeks of gestation [16].

### 2.2. Seating Position of the Dummy

**Figure 1:**
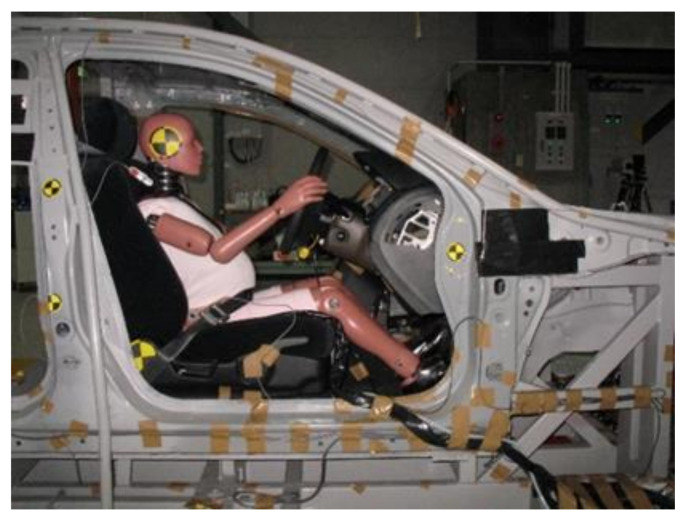
MAMA-2B AF05 ver. sitting in the test setup.

The interior buck used in the present study was fabricated on the basis of the actual vehicle body of the 2003 model of the Honda Accord. The dummy setting was identical in all tests and determined according to the average sitting position as measured for pregnant volunteers having a stature similar to AF05 [16,17]. A pre-test image of the seating position of the dummy in the sled buck is shown in Figure 1. The seating position and posture of the dummy were then fixed with a seat slide position of 70 mm from the full forward position (50 mm forward from the middle position), and a recline of 8.0° from the most upright position (a torso angle of 21° from the vertical line). The referential horizontal distance from the lower rim of the steering wheel to the abdomen was 100 mm.

### 2.3. Test Setup

The Instron Servo Sled Apparatus was used in testing. The seat, steering wheel, and steering column installed in the test setup were the same as those for the vehicle in which the seating positions for volunteers at approximately 30 weeks of gestation were measured. In each test, the dummy was seated in the driver’s seat without a seatbelt. To represent situations of a frontal collision while driving a passenger vehicle, quasi-trapezoid waveforms measured in a flat-barrier test at the time of an impact speed of 13, 26, and 40 km/h were applied to the sled (respectively corresponding to tests 1, 2, and 3).

We used single-stage airbags. An airbag was not deployed in test 1 but deployed in tests 2 and 3. The airbags were ignited in tests 2 and 3 at the time of the onset of the acceleration of the sled buck. These conditions were the same as those in real-world frontal collisions.

### 2.4. Measurements

The overall kinematics of the dummy, such as the trajectory during impact, were examined adopting high-speed video imaging recording at 1000 frames per second. We measured dummy responses, such as the accelerations and distances moved from initial positions for the head, neck, chest, and pelvis. All data were recorded using a high-speed data acquisition system that sampled at 20 kHz and the data were then filtered using a Channel Class 1000 filter. We obtained the pressure on the anterior and posterior abdominal bladder of the dummy (hereafter referred to as abdominal pressure) during impact, which represents the intrauterine pressure for the pregnant women. We checked and calibrated the pressure transducers prior to testing. Klinich et al. identified a correlation between the peak anterior abdominal pressure and adverse fetal outcome from analyses of actual cases [14]. Rupp et al. conducted a series of sled tests using the MAMA-2B dummy and examined the correlation between abdominal pressure on the pregnant dummy and impact speed during vehicle collisions [15]. On the basis of these references, we examined the probability of a negative fetal outcome in each result. To measure deflections of the chest, a system called the Infrared Telescoping Rod for Assessment of Chest Compression (IR-TRACC) was used. This system was mounted inside of the right and left second ribs of the dummy. In addition, from obtained data, we calculated the neck injury criterion (N_ij_), which was developed for the quantitative evaluation of neck injuries and adopted in the automobile collision safety evaluation test as a standard for indicating the degree of neck injury [18,19]. This criterion is based on the correlation of data from dummy tests, cadaver tests, and real-world injuries. Six-axis load cells were mounted at the top of the neck to detect the load and moment around the Y-axis of the neck. This moment corresponded to anterior–posterior movement of the head. The N_ij_ criterion was calculated as N_ij_ = (F_z_ / F_int_) + (M_y_ / M_int_), where F_z_ represents the axial forces in the upper neck (either tension or compression) and M_y_ represents the flexion/extension bending moment at the occipital condyles. A positive F_z_ value indicates tension of the neck while a negative value indicates compression of the neck. The M_y_ value indicates flexion of the neck, with a negative value indicating extension of the neck. F_int_ and M_int_ are critical intercept values used for the normalization of differently sized dummies.

## 3. Results

### 3.1. Kinematics of the Dummy

**Figure 2:**
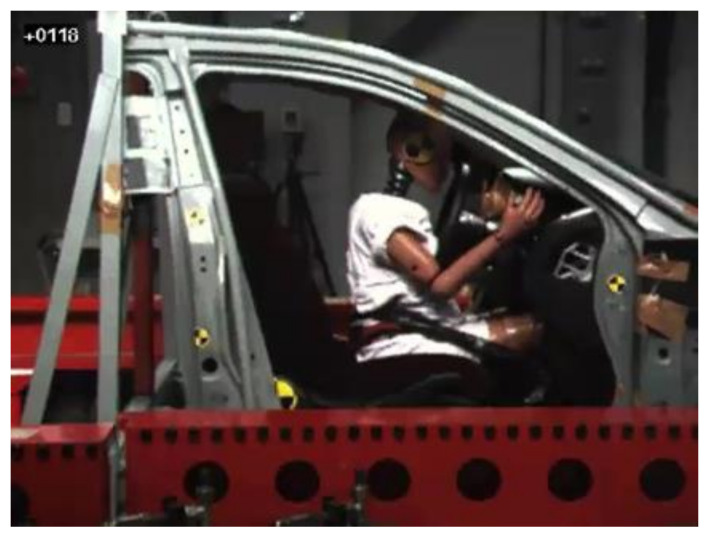
Instant at which the chest of the dummy made contact with the center of the steering wheel in test 1.

**Figure 3:**
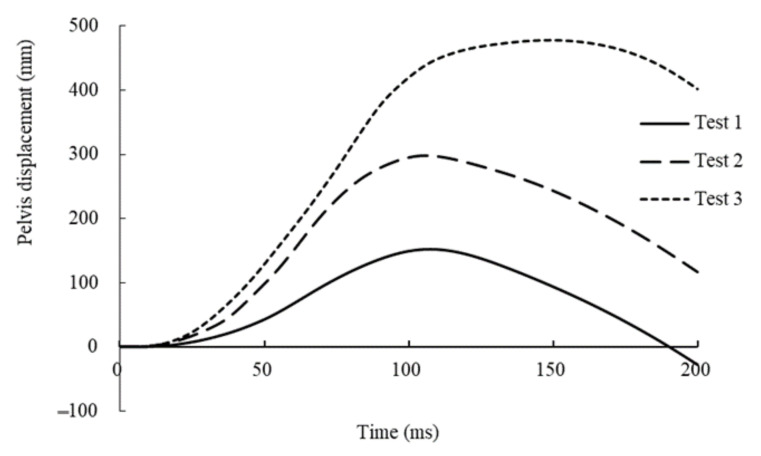
Time courses of pelvis displacement in each test.

In test 1, the dummy moved forward and the abdomen first made contact with the lower rim of the steering wheel at 56 ms from initiation of the impact. The airbag was not deployed. The dummy further moved forward and the chest made slight contact with the center of the steering wheel at 118 ms (Figure 2). Around this time, at 106 ms, the pelvis reached the most forward position (152 mm) from the initial position (Figure 3). The face then made contact with the upper rim of the steering wheel at 141 ms. Finally, the chest of the dummy moved most forward from the initial position (251 mm) at 136 ms from initiation of the impact.

**Figure 4:**
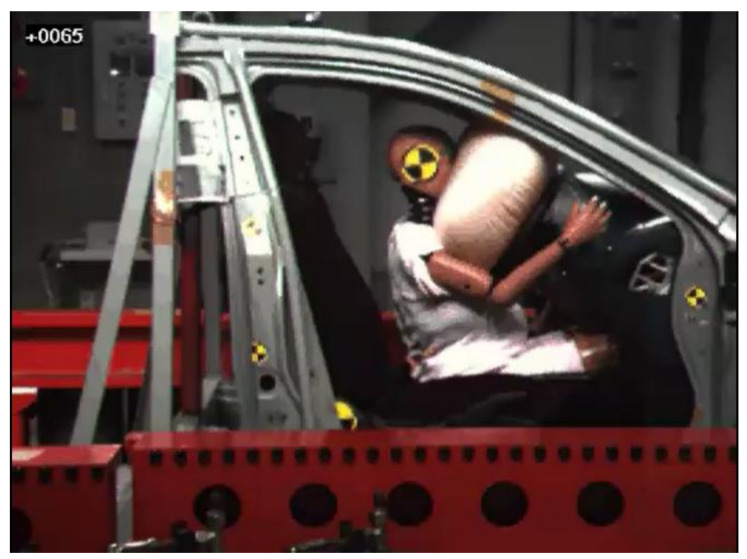
Instant at which the chest of the dummy made contact with the lower rim of the steering wheel in test 2.

In test 2, the dummy moved forward and the head, chest, and abdomen made contact with the airbag at 43 ms from initiation of the impact. The dummy further moved forward and the chest made contact with the lower rim of the steering wheel at 65 ms from initiation of the impact (Figure 4). Finally, the chest of the dummy moved most forward from the initial position (292 mm) at 97 ms from initiation of the impact. Additionally, the pelvis reached the most forward position from the initial position (298 mm) at 105 ms from initiation of the impact (Figure 3).

**Figure 5:**
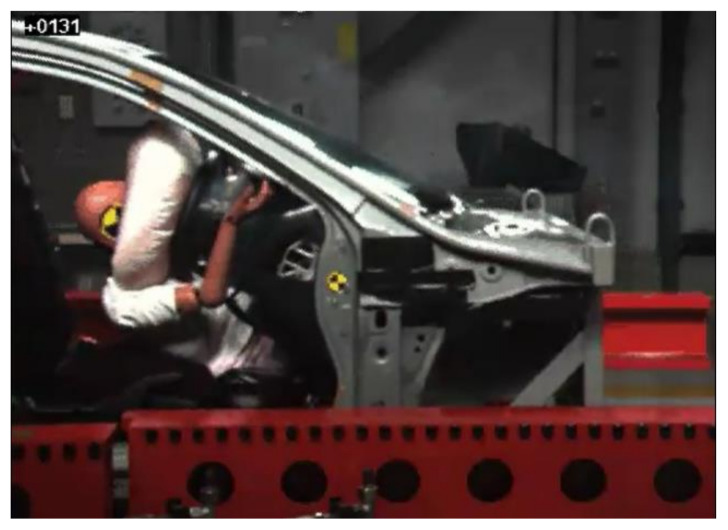
Instant at which the entire dummy’s torso slid under the instrument panel in test 3.

In test 3, the dummy moved forward and the head, chest, and upper part of the abdomen made contact with the airbag at 35 ms from initiation of the impact. The dummy further moved forward and the chest of the dummy made contact with the lower rim of the steering wheel at 69 ms from initiation of the impact, and the pelvis of the dummy subsequently slipped down the seat. Finally, the entire torso of the dummy slid under the instrument panel (Figure 5), and the pelvis reached the most forward position from the initial position (478 mm) at 147 ms from initiation of the impact (Figure 3). Additionally, the chest of the dummy moved most forward from the initial position (383 mm) at 200 ms from initiation of the impact.

### 3.2. Measured Parameters

**Figure 6:**
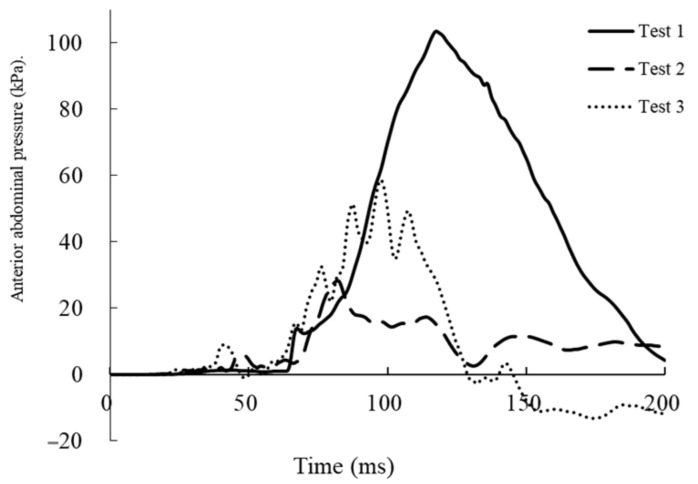
Time course of anterior abdominal pressure in each test.

**Figure 7:**
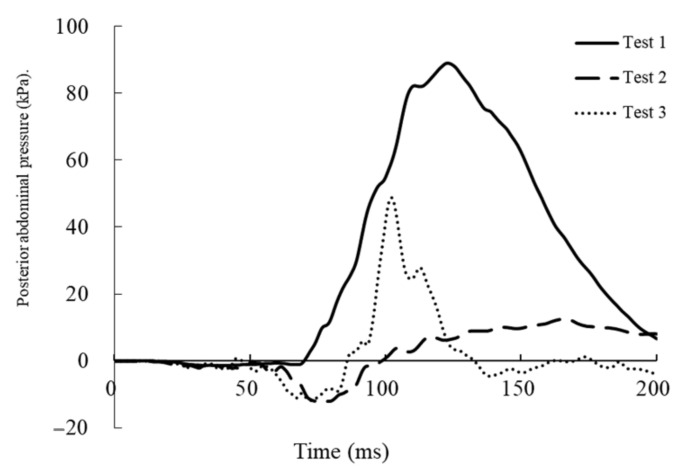
Time course of posterior abdominal pressure in each test.

Table 1 gives the maximum values of measured parameters. Figure 6 and Figure 7 show the time courses of abdominal pressure in each case. In test 1, the maximum abdominal pressure rose to 103.3 kPa in the anterior at 118 ms and to 88.9 kPa in the posterior at 123 ms. The probability of a negative fetal outcome was predicted as about 71% in test 1 and less than 20% in tests 2 and 3. However, maximum values of N_ij_ and chest deflection were low. In test 2, the maximum values of chest deflection were 38.5 mm on the left and 37.9 mm on the right. The value of N_ij_ was 0.36; however, the values of maximum abdominal pressures were lowest among the three tests. In test 3, the values of chest deflections were highest among the three tests and exceeded the predetermined range (>40.9 mm). Additionally, the value of N_ij_ was highest among the three tests (0.61).

## 4. Discussion

We found the cause of negative fetal outcome by analyzing the kinematics of a pregnant woman dummy. To precisely reconstruct a real-world collision involving a pregnant driver, we performed sled tests with MAMA-2B AF05 ver., which has the stature of an average Japanese pregnant women, with the actual seating position measured for Japanese pregnant drivers [16] and with an interior back the same as an actual vehicle body. Our results add novel findings to the work of Rupp. et al. [14].

In this study, the kinematics and mechanisms of injuries of the dummy were different for different collision speeds. Higher impact speeds resulted in longer distances of forward movement of the dummy. However, owing to the interaction between the trunk of the dummy and the airbag or steering wheel, injured body regions were different among the three conditions. In test 1, the dummy moved straightly forward and the abdomen made direct contact with the steering wheel. Therefore, the abdominal pressures of the dummy were markedly higher than those in tests 2 and 3. We considered that the fetal outcome depends on the elevation of the abdominal pressure. From the results of the study, the probability of a negative fetal outcome was predicted as about 71% in test 1 and <20% in tests 2 and 3. Therefore, in test 1, the cause of the negative fetal outcome was a stronger direct force acting on the uterus.

However, in tests 2 and 3 (with a higher impact speed of at least 26 km/h), because the dummy’s abdomen did not make direct contact with the steering wheel, the predicted probabilities of a negative fetal outcome based on peak abdominal pressures were <20%. However, the chest deflection was markedly high because of the direct contact with the steering wheel. The deflection of the sternum is used as injury criterion in current regulatory and consumer tests worldwide to assess the risk of thoracic injury. According to vehicle safety regulations, chest deflections of 2.7, 38.5, and >40.9 mm respectively represent 3.0%, 19.0%, and >21% risks of injuries at an abbreviated injury scale (AIS) greater than 3 [20]. Furthermore, in accordance with a United Nations Regulation (UN-R137), the Japan New Car Assessment Program defined the safety limit of the chest deflection of the AF05 dummy as 34 mm, in a full-lap frontal collision test with a speed of 55 km/h [21]. We therefore supposed that pregnant women would suffer severe chest injuries, such as multiple rib fractures or lung lacerations, when involved in a frontal collision with an impact speed of at least 26 km/h without a seatbelt. In addition, in test 3, the pelvic displacement of the dummy was markedly higher than that in other tests and the pelvis of the dummy slipped down the seat. We supposed that pregnant women would suffer severe pelvic injuries (i.e., pelvic fractures and pelvic viscera injuries) when involved in a frontal collision with an impact speed of at least 40 km/h without wearing a seatbelt. 

We also measured the neck moment of the dummy and calculated Nij [22]. In test 2, because the head of the dummy made contact with the airbag, the neck was extended. In test 3, as the dummy slipped down the seat, the neck was more extended than it was in test 2. A higher Nij value was therefore obtained in test 3 than in test 2. According to the National Highway Traffic Safety Administration, Nij values of 0.12, 0.36, and 0.61 respectively represent 5.0%, 7.0%, and 12.0% risks of injuries at an AIS exceeding 3 and 13.0%, 16.0%, and 21.0% risks of injuries at an AIS exceeding 2 [19]. We supposed that pregnant women might also suffer severe neck injuries when involved in a frontal collision with an impact speed of at least 40 km/h without a seatbelt.

We considered that the mechanism of injuries of the dummy was different at different collision speeds. In test 1, with an impact speed of 13 km/h, the high abdominal pressure of the dummy would lead to a negative fetal outcome. In test 2, with an impact speed of 26 km/h, the severe chest and/or neck injuries would contribute to the fatalities of both the mother and fetus. In test 3, with an impact speed of 40 km/h, in addition to severe chest and/or neck injuries, pelvic injury also contributes to both negative mother and fetal outcomes. Therefore, in tests 2 and 3, although the abdominal pressures were not so high, the negative fetal outcome would be due to the secondary effect of severe or fatal maternal injuries. Therefore, depending on the speed, the cause of negative fetal outcomes must be considered according to the kinematics of the pregnant drivers. Practically, when an unbelted pregnant driver is involved in a frontal vehicle collision, fetal death can occur without any injury to the pregnant women’s abdomen [23]. In these cases, the present results contribute to the decision of forensic pathologists regarding the cause and mechanisms of fetal death.

Because the average longitudinal distance between the steering wheel and the abdomen of a pregnant driver is approximately 100 mm shorter than that for non-pregnant women, the pregnant dummy under each condition of the present study made contact with the steering wheel [16]. The present results reveal that the fetus of the unbelted pregnant driver can suffer fatal injuries even in a low-speed impact. The authors are concerned that a substantial number of pregnant drivers worldwide are at risk of fetal loss because they are not using a seatbelt [24]. Furthermore, Klinich suggested that about 192 fetal losses could be prevented annually if all pregnant women use seatbelts properly in the United States [5]. Therefore, seatbelt use by pregnant women should be promoted for both maternal and fetal safety. Our results emphasize the risk of frontal collision without seatbelts for pregnant women and fetuses from a biomechanical viewpoint.

There are limitations to the present study. First, because the dummy used in this study represented 30 weeks’ gestation, the study determined the cause of negative fetal outcome around 30 weeks of gestation. Therefore, the result does not apply to all pregnant drivers. However, the study found the effect of a protruded abdomen at each collision speed, and the result might be accurate for late-term pregnant women in general. In future work, our result should be confirmed by examining real-world collision cases involving late-term pregnant drivers. Second, we used the peak value of abdominal pressure as the index of negative fetal outcome in this study. We compared values to those in previous reports on drivers in vehicle collisions, and the peak values of the abdominal pressure were thus examined. As different types of force may act on vehicle passengers, other indices of abdominal pressure well correlating to negative fetal outcome could be considered. Third, an interior buck representing a single sedan-type vehicle was used in this study. The geometry of the driver’s seat has no appreciable difference among sedans regardless of the original equipment manufacturer or class of car. However, we understand that sport utility vehicles have a different geometry from sedans, with more upright sitting positions and steering columns, resulting in different outcomes.

## 5. Conclusions

We found the cause of negative fetal outcome by analyzing the kinematics of a pregnant woman dummy. To reconstruct a real-world collision involving a pregnant driver precisely, we performed sled tests with MAMA-2B AF05 ver., which has the stature of the average Japanese pregnant woman, and an interior back the same as the actual vehicle body. Mechanisms of negative fetal outcome were confirmed at different speed of frontal vehicle collisions according to sled tests with a pregnant dummy (MAMA-2B AF05 ver.). At an impact speed of 13 km/h, the high abdominal pressure of the dummy would lead to a negative fetal outcome. Although the abdominal pressures were not so high at a higher impact speed of at least 26 km/h, there would be a negative fetal outcome owing to the secondary effect of severe or fatal maternal injuries. At an impact speed of 26 km/h, severe chest and/or neck injuries would contribute to the fatalities of both the mother and fetus. At an impact speed of 40 km/h, in addition to severe chest and/or neck injuries, pelvic injury also contributes to both negative mother and fetal outcomes. Results suggested that unbelted pregnant drivers suffer severe or fatal injuries to the fetus even in low-speed collisions. These results clarified the detail risk of both fetal and pregnant women’s deaths. Also, this study may contribute to changes in awareness of pregnant vehicle passengers for improving the seatbelt use rate.

## Figures and Tables

**Table 1:** Maximum values of the neck injury criteria, chest deflections, and abdominal pressures under each condition.

Test	Impact Speed (km/h)	Neck Injury Criteria		Chest Deflection				Abdominal Pressure			
				Right		Left		Anterior		Posterior	
		Value	(Time)	Value	(Time)	Value	(Time)	Value	(Time)	Value	(Time)
Test 1	13	0.12	(102 ms)	1.9 mm	(160 ms)	2.7 mm	(148 ms)	103.3 kPa	(118 ms)	88.9 kPa	(123 ms)
Test 2	26	0.36	(100 ms)	37.9 mm	(119 ms)	38.5 mm	(120 ms)	28.3 kPa	(82 ms)	12.5 kPa	(165 ms)
Test 3	40	0.61	(80 ms)	>40.9 mm	(98 ms)	>40.9 mm	(99 ms)	58.6 kPa	(98 ms)	48.7 kPa	(102 ms)

## Data Availability

The data presented in this study are available on request from the corresponding author.
